# A Multi-Band Body-Worn Distributed Radio-Frequency Exposure Meter: Design, On-Body Calibration and Study of Body Morphology

**DOI:** 10.3390/s18010272

**Published:** 2018-01-18

**Authors:** Reza Aminzadeh, Arno Thielens, Sam Agneessens, Patrick Van Torre, Matthias Van den Bossche, Stefan Dongus, Marloes Eeftens, Anke Huss, Roel Vermeulen, René de Seze, Paul Mazet, Elisabeth Cardis, Hendrik Rogier, Martin Röösli, Luc Martens, Wout Joseph

**Affiliations:** 1 Department of Information Technology (INTEC), Ghent University/imec, Technologiepark-Zwijnaarde 15, B-9052 Ghent, Belgium; arno.thielens@ugent.be (A.T.); sam.agneessens@ugent.be (S.A.); patrick.vantorre@ugent.be (P.V.T.); matthias.vandenbossche@ugent.be (M.V.d.B.); hendrik.rogier@ugent.be (H.R.); luc1.martens@ugent.be (L.M.); wout.joseph@ugent.be (W.J.); 2 Department of Epidemiology and Public Health, Swiss Tropical and Public Health Institute, Socinstrasse 57, 4051 Basel, Switzerland; stefan.dongus@swisstph.ch (S.D.); marloes.eeftens@swisstph.ch (M.E.); martin.roosli@swisstph.ch (M.R.); 3 University of Basel, Petersplastz 1, 4001 Basel, Switzerland; 4 Institute for Risk Assessment Sciences (IRAS), Utrecht University, 3508 TD Utrecht, The Netherlands; a.huss@uu.nl (A.H.); r.c.h.vermeulen@uu.nl (R.V.); 5 National Institute for Industrial Environment and Risks (INERIS), Parc Technologique Alata BP2, 60550 Verneuil-en-Halatte, France; rene.de-seze@ineris.fr; 6 Technical Center for Mechanical Industries (CETIM), 60300 Senlis, France; paul.mazet@cetim.fr; 7 Barcelona Institute for Global Health (ISGlobal), 08003 Barcelona, Spain; elisabeth.cardis@isglobal.org

**Keywords:** radio frequency, personal exposure meter, dosimetry, body morphology, calibration, measurement uncertainty

## Abstract

A multi-band Body-Worn Distributed exposure Meter (BWDM) calibrated for simultaneous measurement of the incident power density in 11 telecommunication frequency bands, is proposed. The BDWM consists of 22 textile antennas integrated in a garment and is calibrated on six human subjects in an anechoic chamber to assess its measurement uncertainty in terms of 68% confidence interval of the on-body antenna aperture. It is shown that by using multiple antennas in each frequency band, the uncertainty of the BWDM is 22 dB improved with respect to single nodes on the front and back of the torso and variations are decreased to maximum 8.8 dB. Moreover, deploying single antennas for different body morphologies results in a variation up to 9.3 dB, which is reduced to 3.6 dB using multiple antennas for six subjects with various body mass index values. The designed BWDM, has an improved uncertainty of up to 9.6 dB in comparison to commercially available personal exposure meters calibrated on body. As an application, an average incident power density in the range of 26.7–90.8 μW·m${}^{-2}$ is measured in Ghent, Belgium. The measurements show that commercial personal exposure meters underestimate the actual exposure by a factor of up to 20.6.

## 1. Introduction

Recent advances in wireless communication technologies have had a pivotal role in increasing the number of radio-frequency (RF) electromagnetic sources in the environment. The increase in the number of RF electromagnetic sources is associated with a growing concern about potential harmful health effects of human exposure to RF radiation. These health effects are assessed by introducing limits on the incident RF electromagnetic field strengths, so-called reference levels. An example are those issued by the International Commission on Non-Ionizing Radiation Protection (ICNIRP) [1]. These are quantified in terms of electric field strength or power density incident on the human body. Personal exposure to RF radiation is measured by personal exposure meters (PEMs) [2,3,4,5,6,7,8,9,10,11,12]. These portable devices allow for continuous measurement of the electric field strength at the same location of a subject wearing the device and in different frequency bands for which measurement protocols have been proposed [2,13,14,15]. Besides the above-mentioned advantages of PEMs, a major disadvantage of these devices is their large measurement uncertainty.

Research has shown that PEMs’ measurements are compromised by the presence of human body [3,4,5,6,7,8,9,10,16]. A review of different sources of uncertainty using PEMs for epidemiological studies can be found in [17]. These uncertainties are due to the directivity of the body-worn antennas [5,6,7,9,18,19] or body shadowing in which the body shields part of the EM fields and thus results in an unknown underestimation of the actual EM fields [5,6,7,8,9]. In [4] it was shown that the location of PEMs contributes to the uncertainty of their measurements and results in an underestimation of the incident electric fields. Using numerical simulations in [4], the measurements of PEMs in the range of 98–2450 MHz were estimated at different locations of the body with relative errors up to 140%. In [20], the dependency between the body shadowing and polarization of incident waves has been studied in 2.4 GHz band. The authors of [20] concluded that a vertically polarized antenna results in a higher attenuation due to the body shadowing. Large variations of up to 30 dB and 35 dB in measured power density have been reported using numerical simulations [18] and real measurements [19], respectively. Neubauer et al. [8] reported 90% prediction intervals ($PI_{90}$) of 17 dB around 941 MHz, and 18 dB around 2450 MHz, for PEMs using numerical simulations. Simulated 95% prediction intervals ($PI_{95}$) up to 18.5 dB at 900 MHz and 25.6 dB at 2100 MHz have been reported for a single PEM in proximity of human body [16]. Aminzadeh et al. reported a measured $PI_{50}$ up to 20 dB at 5513 MHz and a simulated $PI_{95}$ of 22.9 dB on the response of a single PEM on the front or back of a human torso [21].

Moreover, PEMs are calibrated in free space while they are used on body. Hence, they measure the electric-fields on the body instead of the actual incident fields. Several studies [5,6,21,22] suggested on-body calibration of PEMs and the use of multiple PEMs on body and a correction factor to compensate for shielding of the body. In order to reduce the measurement uncertainty of PEMs, the concept of a personal distributed exposure meter (PDE) was proposed in [9,23] using on-body calibration measurements in an anechoic chamber. Previously, the design of an exposure meter using multi-detector antennas around the body was also suggested in [22]. A PDE consists of multiple antennas at fixed locations on body, which results in a lower measurement uncertainty due to the variation of antenna location on body. A similar approach is used in [24] for the 60 GHz band. The proposed PDE in [9] has not been used outside the lab. To the best of our knowledge, the previously designed PDEs are limited to single frequency bands e.g., 941 MHz [23] and 2450 MHz [7].

The goal of this study is to, for the first time, design a multi-band Body-Worn Distributed-exposure Meter (BWDM) that simultaneously measures personal exposure to RF fields originating from 11 telecommunication bands. The novelty of the proposed BWDM is to design the first multi-band multi-node wearable exposure meter with synchronized measurements of the nodes at 11 frequency bands. The proposed BWDM is based on the design of textile antenna in [25] and therefore, benefits from high body-antenna isolation. The BWDM is designed and optimized based on the calibration measurements on a human male subject in an anechoic chamber. Moreover, for the first time, a BWDM is calibrated on six people to investigate the effect of human body morphology on its measurement uncertainty. A relation between body mass index (BMI) and recorded personal RF electromagnetic field levels has also been demonstrated for conventional PEMs [26], although never been quantified in previous studies. The BWDM is validated by performing a real measurement campaign in an outdoor environment. This wearable device allows us to assess the personal exposure in real life conditions with less uncertainty. The results will be useful for epidemiological and RF exposure studies that investigate possible effects of exposure to RF radiation on public health. In addition, for the first time, an EME Spy 200 is calibrated on-body in an anechoic chamber to determine its measurement uncertainty. The results are compared to the measurement uncertainty of the proposed BWDM. During future calibration measurements, the influence of the subject’s posture will be considered. Moreover, the application of SAR measurement using the proposed BWDM is part of the future work.

The methodology including the design and calibration of BWDM is described in Section 2. Section 3 presents the results of our study. Section 4 concludes the paper.

## 2. Materials and Methods

Section 2.1 presents the PEMs that are used in this study. The BWDM is constructed using textile antennas and wearable electronics integrated into a garment. The designed antennas and the frequency bands of the BWDM are summarized in Section 2.2. Section 2.3 presents the design of the receiver nodes. The on-body setup is described in Section 2.4, while Section 2.5 explains on-body calibration measurement setup (BWDM in Section 2.5.1, PEMs in Section 2.5.2). The study of body morphology is described in Section 2.6. Section 2.7 provides the details of the outdoor measurement campaign.

### 2.1. Conventional PEMs

Two commercially available PEMs are used in this study: EME Spy 200 (MVG, Brest, France) and ExpoM-RF (Fields at Work GmbH, Zürich, Switzerland). Eleven frequency bands are studied in this paper: The uplink (UL) and downlink (DL) bands of Global System for Mobile Communications (GSM) 900 and 1800 MHz and Universal Mobile Telecommunications System (UMTS) 2100 MHz; Digital Enhanced Cordless Telecommunications (DECT), Wireless Fidelity 2 GHz (WiFi-2G) and 5 GHz (WiFi-5G) and Long-Term Evolution (LTE) downlink bands 800 and 2600 MHz. The detection limit of these exposure meters are 0.01 V/m (EME Spy 200) and 0.05 V/m (ExpoM-RF) for WiFi-5G and 0.005 V/m (both PEMs) for other 10 studied frequency bands (see Table 1).

### 2.2. Textile Antennas

The antenna’s function in the PEM can hardly be underestimated. It is the sensor responsible for capturing the electromagnetic radiation to which the subject is exposed. Its performance and reliability, good or bad, will have severe impact on the reliability of the measurement. That is why careful attention is devoted to the selection and design of the antennas.

To adequately capture the signal, the antenna needs good radiation characteristics, robust on-body performance, and stable figures of merit (primarily gain, impedance matching, and bandwidth). Furthermore, the body-worn nature of the system poses additional challenges, such as bending and compression, but also the need to be able to easily and compactly integrate the antenna into the user’s garments. To achieve this, the choice is made for an antenna constructed from textile materials, based on a topology with high body-antenna isolation.

The antenna design is founded on the topology presented in [25]. It relies on Substrate Integrated Waveguide technology (SIW) to implement an antenna with high-body antenna isolation, which makes the performance robust and reliable. A small form factor is achieved by relying on miniaturization techniques known as “half-mode operation” [27], which makes use of the symmetry of the magnetic field by introducing virtual magnetic walls.

Small size is an asset for body-worn applications and the antenna’s integration potential is further increased by the fact that it is entirely fabricated from textile materials. The conductive parts are realized from copper-coated nylon. The dielectric material composing the antenna substrate is a closed-cell expanded rubber that is commonly found in garments of public service personnel, such as firefighters. To protect the antenna from the surroundings and accidental damaging, the antenna is embedded in a protective textile case consisting of 3D textile materials. A CO${}_{2}$ laser with spot size less than 0.1 μm is used to pattern the textile materials. The different layers are then laminated with the aid of a heat press and thermally activated glue.

**Figure 1:**
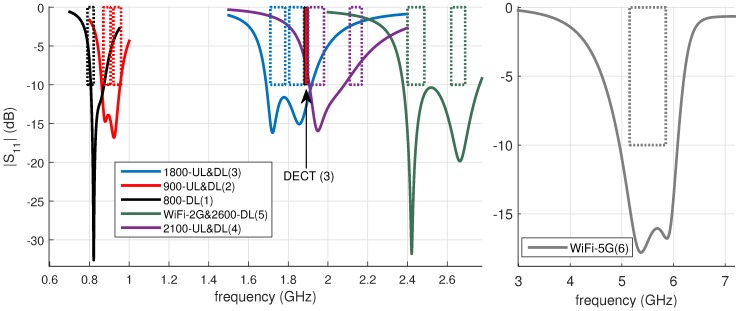
Reflection coefficient of the different antennas, showing good impedance matching for the frequency bands of interest. The numbers in parenthesis are the number of antennas which are listed in Table 1 as antenna Nr.

The antennas are designed and validated by CST microwave studio, where the emphasis is on matching the antenna impedance to the 50 Ω characteristic impedance of the receiver nodes (discussed in the next section), within the frequency band of operation. Therefore, we set as a design goal that $\left|S_{11}\right|$ (the magnitude of the reflection coefficient) remains smaller than −10 dB in a frequency range that is slightly larger than the targeted band of operation. This over-dimensioning is performed to cope with detuning effects that might occur, for example due to the presence of the wearer’s arm in the near field of the antenna. Figure 1 shows the reflection coefficient for the different designed antennas.

Of course, antenna radiation characteristics are also taken into account during the design phase. The main focus is on the maximum realized gain, the pattern shape, and the polarization of the designs. The antenna’s direction of radiation, expressed by the antenna’s radiation pattern, should be directed away from the human body. This makes the antenna sensitive to radiation incident from directions away from the human body. Indeed, when deployed on the body, even an antenna with an omnidirectional radiation pattern in free-space conditions will be unable to receive signals impinging from directions along which signals need to travel through the human body. Such fields will be fully attenuated due to body absorption, putting the human body in a “blind spot”. Therefore, an antenna that only radiates in one hemisphere pointing away from the body is more efficient and less susceptible to variations in human body morphology. In this case, the contribution of waves traveling around the body is negligible w.r.t the signals (energy) received from other directions. The antenna polarization is linear with an axial ratio higher than 5 dB and the antenna gain is between 3 and 5 dBi (along the direction away from the body surface). Table 1 lists dimensions of the designed antennas versus the frequency bands for the BWDM.

### 2.3. Receiver Nodes

**Figure 2:**
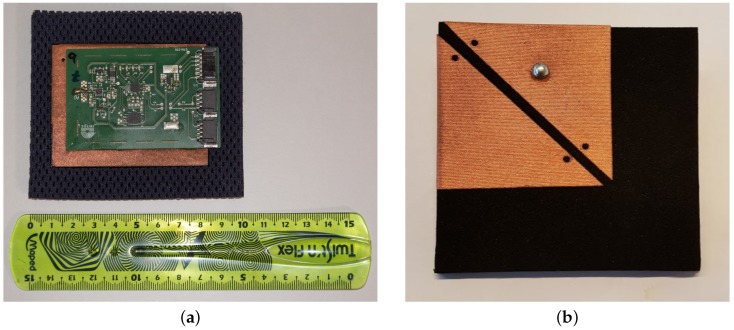
Example of an exposure meter node: (**a**) Back side of the exposure meter node, showing the printed circuit board (PCB) and the ground plane of the antenna under the PCB; (**b**) Front side of the exposure meter node showing the fabricated antenna (resonators) glued to the substrate.

The multi-antenna measurement system consists of 22 autonomously working measurement units for 11 different frequency bands, connected to a common serial bus system. Each exposure meter node is composed of a printed-circuit board, integrated onto a flexible antenna, as displayed in Figure 2. The antenna and compact circuit guarantee an unobtrusive integration into garment, offering a complete measurement and data logging solution.

**Figure 3:**
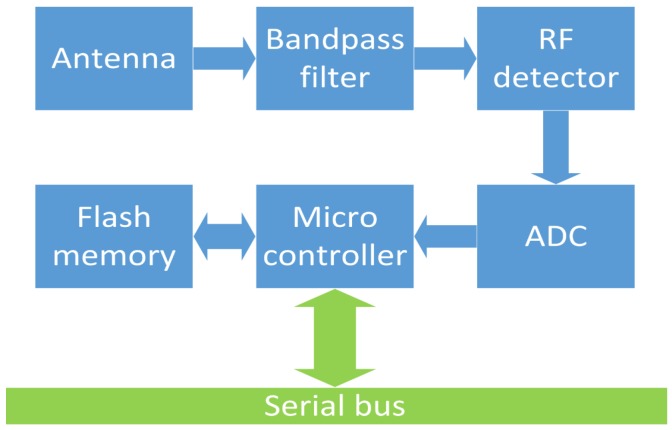
Block diagram of an exposure meter node on the serial bus.

A block diagram of the circuit is shown in Figure 3. This diagram corresponds to one node, attached to the serial bus. The node contains a textile antenna, capturing the impeding signals, which are further filtered to select the desired band of interest for measurement. The filtered signal is then detected by a logarithmic detector chip, operating over a dynamic range of 80 dB. The output of the detector is an analog signal, which is sampled by an analog-to-digital converter, in turn providing the sampled data to the micro controller. The micro controller and its embedded software form the heart of the system, controlling the timing for data logging and calculating minimum, maximum, linear as well as geometric averages. These values are stored into flash memory each second.

In total, 22 custom nodes for different frequency bands are connected to the bus system, integrated into the jacket. The bus system allows reading out the data of the flash memory for all the nodes automatically. The bus communication is controlled by a master unit, which can be connected to a laptop computer. This master unit is portable. It monitors the correct operation of the nodes during measurement campaigns. Additionally, the user can synchronously record time stamps in all the slave nodes by pressing a button on the master unit.

### 2.4. On-Body Setup

**Figure 4:**
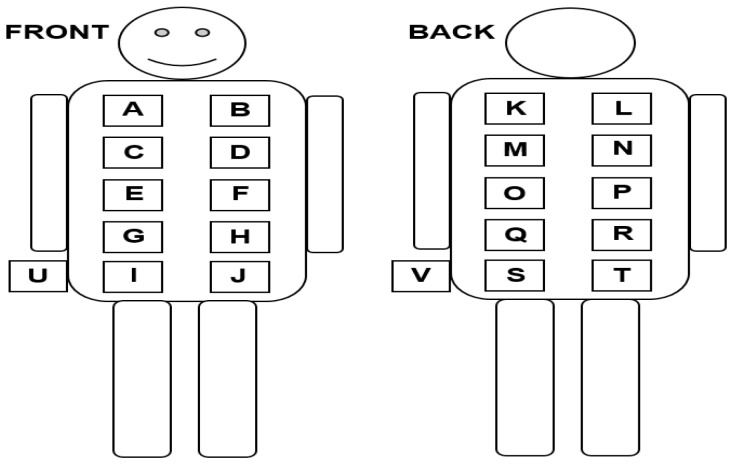
The proposed nodes’ locations on the front and back of the torso.

Twenty two potential locations to position the antennas on body are examined. Only the torso of human body and hips are considered for the deployment of the antennas on body. This was motivated by the fact that, on these areas, the antenna performance would be less affected by the user movements. Both the front and back of the torso are divided into 22 locations (are labeled as A-T, U and V) as shown in Figure 4. For each frequency band, two antennas are placed on diametrically opposite locations on the torso [28] or on the hips. This requires 2244 measurements: 11 (bands) × 51 (positions on torso/hips: A to V) × 4 (2 polarizations of transmitter and receiver antennas). This was more feasible in a realistic time space. Therefore, a set of locations on body are selected randomly for each frequency band covering all the proposed locations on body and thus 120 measurements are performed. The position and polarization of the nodes are optimized using on-body calibration measurements (see Section 2.5) on a 28-year old male subject (which is denoted as Sb-1). Only the standing posture is considered here, the influence of posture is part of future work.

Two PEMs are placed on the right (EME Spy 200) and left (ExpoM-RF) lateral hips (just below the waist) of Sb-1, based on whom the BWDM is designed and calibrated (see Figure 4). These are the only potential locations on the body which are not affected by the presence of the antennas in the BWDM. A belt has also been used to prevent movements of the PEMs during the measurements.

### 2.5. Calibration Measurements

The goal of calibration is to compare actual fields with and without the body and to determine the measurement uncertainty of the BWDM as well as EME Spy 200 and ExpoM-RF. Section 2.5.1 and Section 2.5.2 present the on-body calibration of BWDM and the PEMs, respectively.

#### 2.5.1. On-Body Calibration of BWDM

The calibration procedure is proposed to simultaneously determine an optimized location for each antenna per frequency band as well as an effective on-body antenna aperture. For each frequency band, two receiver nodes (RX) are diametrically placed on locations *i* on the front and back of the torso since this leads to a lower measurement uncertainty [6,7].

First, the subject (Sb-1) is placed on a rotational platform in the far field of a transmitting horn antenna (TX) in an anechoic chamber. The subject is rotated over 360${}^{\circ }$ at an angular speed of 2${}^{\circ }$ per second around his axis perpendicular to the ground floor of the chamber. During each rotation, the receiver nodes register the received power on body at a sampling rate of 1 Hz. This procedure is repeated for both vertical (V) and horizontal (H) polarizations of the TX, resulting in the received powers $P_{r,ij}^{V}\left(\varphi \right)$ and $P_{r,ij}^{H}\left(\varphi \right)$, respectively. The first step is repeated to examine both V and H orientations of the RX. In each frequency band *j*, the TX emits a constant power in the range of 20–60 mW. Second, using a Narda (NBM-550) isotropic field probe, the free-space incident power densities $S_{inc,j}^{free,V}$ and $S_{inc,j}^{free,H}$ are measured along a line at the subject’s location 54–202 cm above the rotational platform for both V- and H polarizations of the TX, respectively. This step provides the incident power densities in free space (without the body) at the location of the subject. Third, the received powers on body (first step) and the free-space incident power densities (second step) are used to determine the arithmetic or geometric averaged AA of the BWDM (over front and back) for any realistic polarization:

(1) $$AA_{ij}\left(\theta =90^{\circ },\varphi ,\psi \right)=\frac{P_{r,ij}^{H}}{S_{inc,j}^{free,H}}cos^{2}\left(\psi \right)+\frac{P_{r,ij}^{V}}{S_{inc,j}^{free,V}}sin^{2}\left(\psi \right),$$

where *i* is the antenna on the front or on the back and ψ is the polarization of an incident electric field. It must be noted that, in the anechoic chamber used for this study, it is not possible to measure all incident polar angles. In a real multipath environment no assumption can be made about incident polarization. In order to determine $AA_{i}\left(\varphi ,\psi \right)$ for any realistic polarization, 1000 ψ samples are drawn from a uniform distribution in the range of $\left[0,2\pi \right]$ that results in a certain distribution for the on body antenna aperture. This is repeated in a loop (bootstrap) with 100 iterations to assess the variation on analysis. The 16%, 50% and 84% percentiles of this distribution are determined and are denoted as $p_{16}$, $p_{50}$ and $p_{84}$, respectively. The median ($p_{50}$) of the distribution is used to convert the measured received powers on the nodes to incident power densities $S_{inc,j}^{meas}$:

(2) $$S_{inc,j}^{meas}=\frac{P_{r,ij}^{meas}}{p_{50}\left(AA_{ij}\right)},$$

where $P_{r,ij}^{meas}$ is the received power on each node at location *i* and band *j*. The other percentiles are used to estimate the 68% confidence interval ($CI_{68}$) of the on-body aperture:

(3) $$CI_{68,ij}=\frac{p_{84}\left(AA_{ij}\right)}{p_{16}\left(AA_{ij}\right)},$$

From (3) it is clear that minimizing $CI_{68}$ will minimize the uncertainties as well. Therefore, a minimal $CI_{68}$ is determined as the minimum of the $CI_{68}$’s on measurements with a V- or H-polarized node placed on location *i* and measuring in the frequency band *j*.

#### 2.5.2. On-Body Calibration of Conventional PEMs

The goal of on-body calibration is to compare the measurement uncertainty of the designed BWDM with those of the conventional PEMs. For the first time, an EME Spy 200 is calibrated on body and its measurement uncertainty is assessed in terms of $CI_{68}$. On-body calibration measurements are performed in the anechoic chamber, similar to the steps explained in Section 2.5.1 for BWDM. During each rotation, the incident electric field $E_{kj}^{body}$ was measured by both PEMs, at a sample rate of 0.25 Hz for every angle of φ, resulting in a distribution for the recorded electric fields by each PEM and for both polarizations of the TX. Next, the response $R_{k}$ of the PEMs is determined for any realistic polarization in a loop with 100 repetitions:

(4) $$R_{k}\left(f_{j},\varphi \right)={\left(\frac{E_{kj}^{body}\left(f_{j},\varphi ,\theta =90\right)}{E^{free}\left(f_{j}\right)}\right)}^{2},$$

where *k* is the location of PEMs on body (right or left hip), $f_{j}$ is the center frequency of each studied band and $E^{free}$ is the free space incident electric field measured by the Narda probe (see Section 2.5.1). The 16%, 50% and 84% percentiles of this distribution are determined. They are denoted as $p_{16}$, $p_{50}$ and $p_{84}$, respectively. The median ($p_{50}$) of the distribution is used to correct the measured data on these PEMs $E^{meas}$ for the effect of human body:

(5) $$E^{inc}\left(f_{j}\right)=\frac{E_{kj}^{meas}\left(f_{j}\right)}{\sqrt{p_{50}\left(R_{k}\left(f_{j},\varphi \right)\right)}},$$

### 2.6. Study of Body Morphology

Using the minimal $CI_{68}$ values for each frequency band, the location of each node was optimized on Sb-1. Once the locations of nodes are finalized on the vest, the BWDM is worn by five more subjects (Sb-2 to Sb-6). The same on-body calibration is performed to determine the effect of body morphology on measurement uncertainty of the BWDM in terms of $CI_{68}$. Table 2 lists the characteristics of the people that participated in this study. The presence of different body morphologies on measured uncertainty of the BWDM is reported in terms of the standard uncertainties of the on-body antenna apertures ($AA_{ij}$). Such uncertainties correspond to the standard deviations of the AA’s distributions. However, Thielens et al. [6,7] demonstrated that the distributions of $AA_{ij}$ can be asymmetric. Therefore, we defined the upper standard uncertainty $s_{up,j}$ and lower standard uncertainty $s_{low,j}$ for the optimized design of the BWDM worn by the six subjects:

(6) $$s_{low,j}=1-\frac{p_{50}\left(AA_{j}\right)}{p_{84}\left(AA_{j}\right)}$$

(7) $$s_{up,j}=\frac{p_{50}\left(AA_{j}\right)}{p_{16}\left(AA_{j}\right)}-1$$

The uncertainty caused by the presence of human body on the total power density is defined as the sum of upper and lower uncertainties for *j* bands:

(8) $$s_{low/up,total}=\sqrt{\sum_{j=1}^{10}{\left(s_{low/up,j}\right)}^{2}}$$

### 2.7. Application: Real Measurements in Outdoor Environments

**Figure 5:**
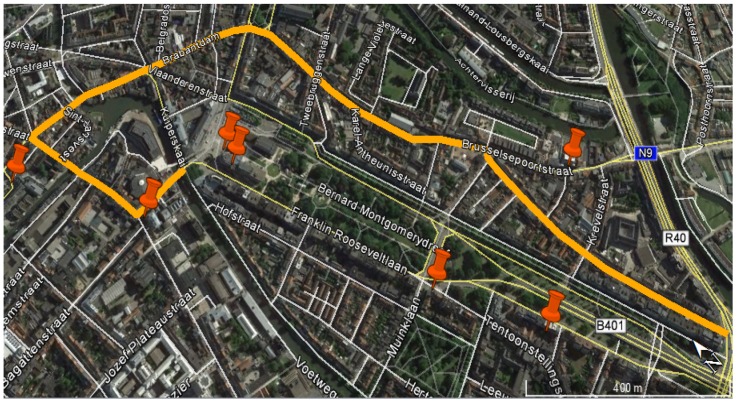
The predefined walk in the center of Ghent, Belgium. The thick line indicates the route. The orange place markers indicate the location of antennas. (The location of antennas are extracted from [29]).

The purpose of this outdoor measurement campaign is to demonstrate the applicability of the BWDM for real-life exposure measurements and to assess the measurement uncertainty. The test person (Sb-1) equipped with the BWDM and two commercial exposure meters (EME Spy 200 and ExpoM-RF on the right and left hips, respectively) walks along a predefined path (see Figure 5) in a residential area close to the center of Ghent, Belgium. The route (thick line) is approximately 2.5 km long and lasts 33 min. During the measurements the received power on each node is registered and the incident power density is calculated for each frequency band using the determined on-body AA from the calibration measurements.

## 3. Results and Discussion

### 3.1. On-Body Calibration of Conventional PEMs

Table 3 lists the results of the on-body calibration of PEMs on Sb-1 in the anechoic chamber. The median *R* for the EME Spy 200 is in the range of 0.12 (DECT) to 0.73 (2600-DL). Also the range of $CI_{68}$ for the EME Spy 200 is 8.99 (2600-DL) to 13.68 dB (WiFi-5G). The results clearly indicate that the EME Spy 200 on the right hip underestimates the measured electric fields by a factor of 1.1 to 2.88. For ExpoM-RF, the median *R* is in the range of 0.26 (2100-DL) to 4.59 (WiFi-5G), which is an under/over estimation of the actual electric fields by a factor of 0.4 to 1.9. The value of $CI_{68}$ is in the range of 8.5 (WiFi-2G) to 12.6 dB (900-DL). The on-body calibration of ExpoM-RF on the left hip of Sb-1 and on the hip of another male subject in [30] shows that there is a difference of 0.35 to 8.7 dB for the $CI_{68}$ of their responses due to different body morphologies.

### 3.2. On-Body Design and Calibration of the BWDM

The optimized design of the BWDM is summarized in Table 4. The optimized location and polarization of nodes that provide the minimal $CI_{68}$ of the on-body antenna aperture for Sb-1 are presented, composing the optimal configuration of the nodes on body. For each frequency band, the median AA and $CI_{68}$ are determined for the arithmetic and geometric averages over two nodes on the front and back. The results show that geometric averaging over the two nodes provides a lower $CI_{68}$. For example, the geometric average over nodes $A^{V}$ (location: A, vertical polarization) and $S^{H}$ (location: S, horizontal polarization) in the 1800-UL band yields a lower median $CI_{68}$ of 3.8 dB. Therefore, the geometric average over front and back is considered to determine the geometric on-body antenna aperture for each subject. The BWDM exhibits an AA range of 0.33 cm${}^{2}$ (WiFi-5G) to 12.71 cm${}^{2}$ (900-DL) and $CI_{68}$ of 3.3 dB (2100-DL) to 5.67 dB (2600-DL). The results show that the designed BWDM provides relatively low $CI_{68}$ values. Moreover, the $CI_{68}$ values of the BWDM are compared to the $CI_{68}$ of PEMs obtained from on-body calibration of PEMs on the same subject (Sb-1). Results are listed in Table 4. The PEMs yield relatively higher uncertainties: for example, the EME Spy 200 has higher $CI_{68}$ values than the BWDM, being a 3.3 dB (58.5%) increase for 2600-DL to a 9.6 dB (291%) increase for 2100-DL. The ExpoM-RF exhibits a $CI_{68}$ which is 3.6 dB larger (63.5%) for 2600-DL to a 8.1 dB higher (214.5%) for 1800-UL, in comparison to the BWDM on the same subject.

**Figure 6:**
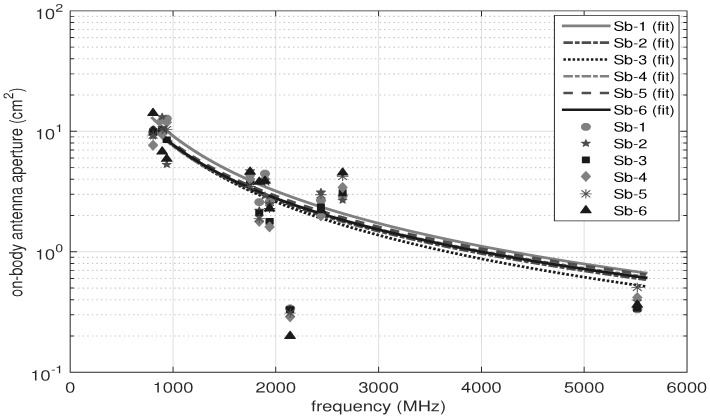
The fitted models to the on-body AA values for six people. Vertical axis: frequency (MHz), horizontal axis: AA (cm${}^{2}$).

Figure 6 depicts the on-body antenna apertures of the BWDM for the six subjects as a function of frequency. The results show that increasing the frequency decreases the AA values. The following function is fitted to the AA values per person as a function of frequency:

(9) $$AA=a\left(10^{14}\right)f^{b}$$

where AA is the on-body antenna aperture (cm${}^{2}$), f is the frequency (Hz) and *a* and *b* are the fit coefficients. The goodness of fit is evaluated by $R^{2}$ and is in the range of 0.72 to 0.91. The *a* and *b* are in the range of 1.73 to 3.3 and −1.47 to −1.57, respectively. In theory, AA is proportional to the wavelength ($\lambda^{2}$) and, hence, to $1/f^{2}$ [31]. This (b=−2) is for antennas that have the same directivity. The values of *b* obtained in this paper are slightly different than −2, which is due to the influence of body on the antennas on body and also the different directivity. Moreover, no obvious relationship between the BMI and *a* and *b* parameters is found. Either increasing or decreasing the BMI showed variation of the fit parameters. For subjects with a similar height exhibited a similar value of *a* and *b*: for example, 1.73 vs. 1.87 for *a* and −1.1 vs. −1.4 for *b* (Sb-4 vs. Sb-5).

### 3.3. Study of Body Morphology

**Figure 7:**
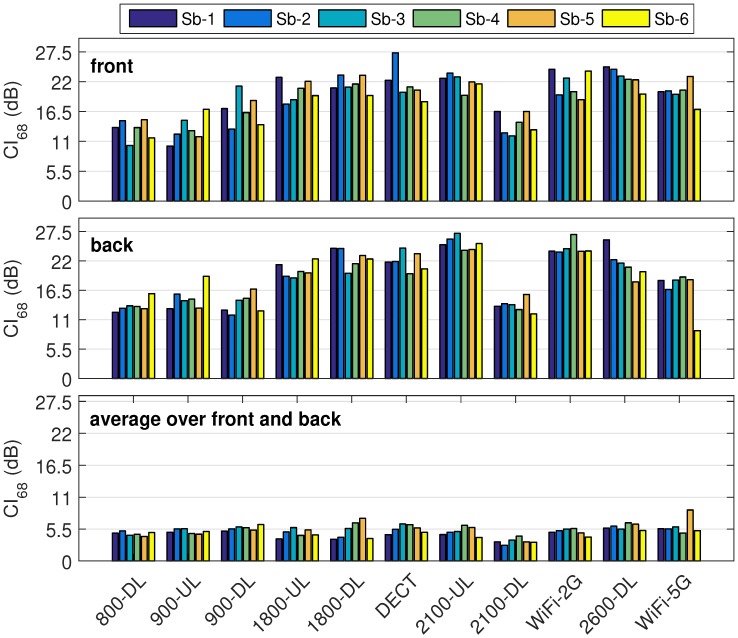
Median 68% confidence interval of the on-body antenna aperture for each person per frequency band.

Figure 7 shows the $CI_{68}$ (median $CI_{68}$ obtained using the bootstrap with 100 repetitions) of the on-body antenna aperture for 6 subjects and 11 frequency bands. The value of $CI_{68}$ is in the range of 10 dB (900-UL Sb-1) to 27.3 dB (DECT, Sb-2) for the nodes on the front. For nodes on the back of torso, the minimum and maximum $CI_{68}$ are 11.8 dB (900-DL, Sb-2) and 27.1 dB (2100-UL, Sb-3), respectively. Using the geometric averaging over each pair of nodes on the front and on the back of torso in each frequency band, the range of $CI_{68}$ decreases and is in the range of 2.69 dB (2100-DL, Sb-2) and up to 8.8 dB (WiFi-5G, Sb-5). For the optimized BWDM, this is an improvement of up to 22 dB in measurement uncertainty with respect to single nodes on the front or back of the torso. The results show that, for different subjects (different BMIs), the variation for single nodes is higher. The range is 4 to 6.7 dB (factor of 4.6), for the nodes on the front, and 3.7 to 7.8 dB (factor of 6), for the nodes on the back. Calibration measurements demonstrate that the geometric average over the nodes on the front and back, reduces the variation of $CI_{68}$ for all different subjects in the range of 1.2 dB (1800-UL) to 3.6 dB (1800-DL) for 11 frequency bands.

**Figure 8:**
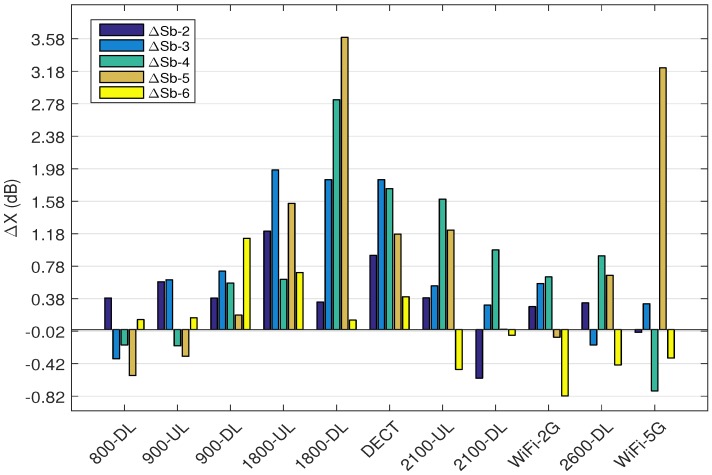
difference in $CI_{68}$ for subjects Sb-2 to Sb-6 with respect to Sb-1.

Figure 8 depicts the difference in $CI_{68}$ values (ΔX) for subjects Sb-2 to Sb-6 with respect to Sb-1 (ΔX $\;=CI_{68,X}-CI_{68,Sb-1}$; X = [Sb-2,...,Sb-6]). Since the location and polarization of the nodes are optimized for Sb-1, the ΔX is calculated considering Sb-1 as the reference. The ΔX is expected to be positive for Sb-2 to Sb-6, which is true if Sb-1 has the lowest $CI_{68}$ for all the bands. The results show that ΔX is positive for 40 out of 55 (72.7%) measurements, which is acceptable. Among the 28% of negative ΔX’s, the largest difference is for subject Sb-6, which has only 0.82 dB lower $CI_{68}$ compared to Sb-1 (WiFi-2G). For Sb-2, the minimum and maximum ΔSb-2 are 0.6 dB (2100-DL) and 1.2 dB (1800-UL), respectively. Sb-3, has a minimum 0.3 dB (800-DL) and maximum 1.9 dB (1800-UL). The minimum and maximum ΔSb-4 are 0.2 dB (900-UL) and 2.8 dB (1800-DL), respectively. For subject Sb-5, 0.5 dB (800-DL) is the minimum and 3.6 dB (1800-DL) is the maximum difference with respect to Sb-1. The minimum ΔSb-6 is 0.8 dB (WiFi-2G) and 1.1 dB (900-DL) is the maximum. Subjects Sb-2 (BMI 23.9 kg/m${}^{2}$) and Sb-3 (BMI 25.5 kg/m${}^{2}$) with similar heights (178 cm) have the maximum ΔX with respect to subject Sb-1 for 1800-UL. Among the subjects, Sb-4 (BMI 33.2 kg/m${}^{2}$) and Sb-5 (BMI 23.3 kg/m${}^{2}$) have the most similar heights (169 and 167 cm, respectively) and also the maximum ΔX at 1800-DL. It may be concluded that for both 1800 MHz UL and DL bands $CI_{68}$ is a function of height rather than the BMI.

Furthermore, the maximum ΔX decreases (ΔSb-5 > ΔSb-4 > ΔSb-3 > ΔSb-2 > ΔSb-6) with increasing height of the person ($h_{Sb-5}<h_{Sb-4}<h_{Sb-3}<h_{Sb-2}<h_{Sb-6}$). Considering subjects with similar heights, for 900-UL, (Sb-2, Sb-3) and (Sb-4, Sb-5) have a similar ΔX with respect to Sb-1, (0.58, 0.61 dB) and (0.2, 0.3 dB), respectively. Sb-2, with a similar BMI to Sb-1 (difference of 0.3 $kg/m^{2}$), has a constant ΔSb-2 of about 0.3 dB in 6 out of 11 bands (800-DL, 900-DL, 1800-DL, 2100-UL, WiFi-2G and 2600-DL). For DECT, Sb-3 and Sb-4 with largest BMI values have the largest ΔX values of 1.8 and 1.7 dB, respectively. Sb-4 and Sb-5 with a similar height have ΔSb-4 of 1.6 dB and ΔSb-5 of 1.2 dB. For DECT and 2100-UL bands, the values of ΔSb-4 and ΔSb-5 are similar (1.7 and 1.6 dB for Sb-4; and 1.1 and 1.2 dB for Sb-5). This might be due to the cross talk. For 2100-DL, subjects Sb-5 and Sb-6 with a similar BMI to Sb-1 have ΔSb-5 of 0.005 dB and ΔSb-6 of 0.06 dB. Sb-6 with a similar height to Sb-1, has a difference of maximum 0.7 dB (except 900-DL: 1.2 dB). According to the results, except 1800-DL and WiFi-5G, the maximum deviation for all the subjects is less than 2 dB for all frequency bands.

Table 5 lists the 68% confidence interval of the on-body antenna aperture for different subjects and 11 frequency bands. For Sb-1, the $CI_{68}$ ranges from 3.2 (2100-DL) to 5.6 dB (2600-DL). The $CI_{68}$ values for Sb-2, range from 2.6 (2100-DL) to 6 dB (2600-DL). For Sb-3, the $CI_{68}$ is in the range of 3.6 (2100-DL) to 6.3 dB (DECT). Sb-4 has $\left(CI_{68}\right)$ in the range of 4.2 (2100-DL) to 6.5 dB (2600-DL). Sb-5 and Sb-6 have a minimum $CI_{68}$ of 3.3 and 3.2 dB (2100-DL) and maximum 8.8 dB (WiFi-5G) and 6.2 dB (900-DL), respectively. Based on the results, the measurement uncertainty in different frequency bands, in terms of $CI_{68}$, can be reduced when 2 antennas are placed on body. These results are much (7 to 10 dB) lower than the $CI_{68}$ of a commercial exposure meter (ExpoM-RF 64), calibrated on the left hip of a male subject [30]. The results are consistent over all subjects. In addition, ExpoM-RF’s calibration on Sb-1 shows 3.6-8.1 dB higher uncertainty compared to the calibrated BWDM on the same subject (see Table 4). This reduction is due to the negative correlation between the nodes on the front and the back of torso. Moreover, this reduction is associated with geometric averaging over measurements of the same quantity. The small standard deviation shows the reliability of the data and also the implemented bootstrap method to determine the on-body antenna aperture for each subject.

The median on-body AA values determined from calibration measurements on six subjects as well as the uncertainties due to the presence of human body in each frequency band are presented in Table 6.

The AA values range from 0.2 cm${}^{2}$ (2100-DL, Sb-6) to 14.1 cm${}^{2}$ (800-DL, Sb-6). Increasing the frequency (except 2100-DL) results in less variation in the AA values for different subjects since AA is smaller for higher frequencies (see Table 4). The antenna apertures depend on the directive gain of the used antenna, the antenna efficiency and the square of the wavelength of the received signals. The smaller antenna aperture at 2100-DL is due to the electrical distance that affects the antenna performance in presence of human body. The upper and lower standard uncertainty shows that the distribution of on-body AA is asymmetric for all the subjects at each frequency band. This is in agreement with the results presented in [6,7]. The lower and upper boundaries of the uncertainty caused by the presence of human body on the total power density $s_{low,total}$ and $s_{up,total}$ for subjects with a similar BMI (Sb-1, Sb-2, Sb-5 and Sb-6) are comparable. For example Sb-2 and Sb-5 have a different uncertainty of 3.2% for $s_{low}$ (42.6% vs. 45.8%) and 2.4% for $s_{up}$ (85.1% vs. 87.5%). This means that, despite the asymmetric shape of the AA distributions, for subjects with a similar BMI, the corresponding boundaries have less variations.

### 3.4. Application: Real Measurements in Outdoor Environments

**Figure 9:**
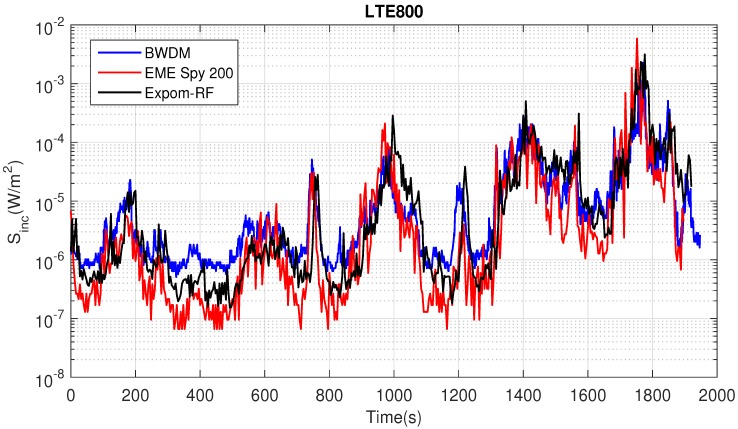
An example of the incident power densities measured during the walk for 800-DL.

Figure 9 shows an example of the measured incident power density $S_{inc}$ for 800-DL for the three devices during the walk.

Both the EME Spy 200 and ExpoM-RF underestimate the exposure to 800-DL signals during the walk. The measurement uncertainty of the PEMs might depend on their location on the body [7] and a lower uncertainty might be achievable for the PEMs if they would be placed on another location on the body. However, a sensitivity study of the PEMs’ location on the body was not possible in this study, due to BWDM, which covers most of the subjects’ torso. A potential way to get an exact comparison between the PEMs uncertainty and the BDWM would be to place the PEMs on every potential location on the body where the BWDM nodes are placed (see Figure 4) and perform a calibration following the procedure described in Section 2.5. However, since we only performed measurements with the PEMs on the hips (not in other potential configurations). We limited ourselves to only calibrating on those on-body locations.

Table 7 lists summary statistics of the measurement for three cellular technologies 2G, 3G and 4G including 800-DL, 900-DL, 1800-DL and 2100-DL bands. For the BWDM, censoring occurs at 2100-DL for which only 4.2% of the data are censored. This band, has the smallest AA and therefore, results in a higher on-body detection limit. For the rest of the bands, the BWDM registered no censored data due its low detection limits. For the EME Spy 200, up to 14% of the measurement data are censored while for the ExpoM-RF, 1.03% of the measurements (at 1800-DL) are censored. In order to calculate the summary statistics, the Robust Regression on Order Statistics (ROS) [2] is applied to the measurements. For the BWDM, the mean measured power densities are in the range of 26.7 μW/m${}^{2}$ (800-DL) to 90.8 μW/m${}^{2}$ (900 DL). The large standard deviation is due to the city environment where several buildings with different heights are present. For the EME Spy 200, the mean power density ranges from 4.41 μW/m${}^{2}$ (1800-DL) to 60.1 μW/m${}^{2}$ (900-DL). ExpoM-RF registered mean power densities in the range of 14.53 μW/m${}^{2}$ (1800-DL) to 151.5 μW/m${}^{2}$ (900-DL). All the three devices measured the maximum mean power density for 900-DL band (2G). For the BWDM, the median $S_{inc}$ is in the range of 3.21 μW/m${}^{2}$ (800-DL) to 29 μW/m${}^{2}$ for 2100-DL. Both PEMs registered the minimum $p_{50}$ of $S_{inc}$ for 1800-DL (EME Spy 200: 0.59 μW/m${}^{2}$, ExpoM-RF: 1.62 μW/m${}^{2}$) and the maximum median $S_{inc}$ for 900-DL (EME Spy 200: 12.26 μW/m${}^{2}$, ExpoM-RF: 34.77 μW/m${}^{2}$). The results are comparable to the previous studies. For example, using a single-band PDE on body, Bhatt et al. reported a median $S_{inc}$ in the range of 0.51 to 51.24 μW/m${}^{2}$ for a number of residential areas in Ghent, Belgium for 900-DL [32]. In this study, the BWDM measured a median $S_{inc}$ of 28 μW/m${}^{2}$ in the same band. The difference may be due to the diverse measurement locations in the city. In this study, the EME Spy 200 registered median $S_{inc}$ of 1.28 in 800-DL band, while, Hardell et al. reported a median power density of 9.5 μW/m${}^{2}$ in Stockholm, Sweden [33]. The higher exposure level in Stockholm is due to the LTE base stations. To the extent of our knowledge, in this paper, for the first time, LTE signals are measured in a real environment using a BWDM. All the measured values are below the issued reference levels (2 W·m${}^{-2}\leq S_{inc}\leq 10\;W\cdot$m${}^{-2}$) by ICNIRP [1] for the general public.

A ratio is defined (see Table 7) for of the median values of BWDM to EME Spy 200 and ExpoM-RF. According to the measurements, EME Spy 200 underestimates the median actual incident fields by a factor of 2.28 (900-DL) to 20.67 (1800-DL). This value is in the range of 1.68 (800-DL) to 9.92 (2100-DL) for the ExpoM-RF. The only exception is 900-DL for which the ExpoM-RF measured 1.2 times higher median $S_{inc}$ than the BWDM. This might be due to the position of Expom on body during the walk in a way that the ExpoM-RF is faced toward a base station and thus measured higher values. Comparing the median $S_{inc}$ measured by BWDM, 800 -DL (4G) has the lowest exposure level, while the exposure to 2G signals (900 and 1800 MHz downlinks) are 3.8 to 8.7 times higher than 4G, 3G is 9 times higher than 4G and 2.3 times higher than 2G (1800-DL).

## 4. Conclusions

For the first time, a multi-band body-worn distributed-exposure meter (BWDM) is proposed for simultaneous on-body measurements of the incident power density in 11 telecommunication bands. The BWDM is designed and calibrated on a male human subject, in an anechoic chamber. The optimized location of 22 nodes covering 11 frequency bands is determined on the front and on the back of the torso. The optimized BWDM is also calibrated on five more subjects in order to study the effect of human body morphology on the measurement uncertainty of the designed BWDM. The uncertainty is quantified as the 68% confidence interval of the on-body antenna aperture obtained during calibrations. It is shown that using multiple antennas improved the uncertainty up to 22 dB with respect to single nodes for all subjects in all frequency bands. We also demonstrated that, using single antennas, the variation on $CI_{68}$ for the six people in this study was about 9.3 dB. This value reduced to the range of 1.2 to 3.6 dB for all the subjects, which is 5.7 dB improvement. Except for 1800-DL (3.6 dB) the maximum variation on the $CI_{68}$ of subjects of this study is limited to below 2 dB. The designed BWDM has an improved $CI_{68}$ of 9.6 dB compared to $CI_{68}$ of commercially available PEMs calibrated on body. Using the proposed BWDM and two PEMs, fields along an outdoor route are measured in Ghent, Belgium including 800, 900, 1800 and 2100 MHz downlink bands. The BWDM measured a mean power density in the range of 26.7 to 90.8 μW/m${}^{2}$, which are below the issued reference levels by ICNIRP. The results show that commercial PEMs underestimate the actual incident power densities by a factor of 1.6 to 20.6. Moreover, the measured exposure to 2G and 3G signals are 3.8 to 9 times higher than the 4G signals. The study of the subject’s posture and application of SAR measurement will be part of the future work.

## Figures and Tables

**Table 1:** Parameters of the designed textile antennas. f${}_{c}$: center frequency. Antenna Nr: antenna number; these numbers are shown in parenthesis in Figure 1.

Band Name	Range (MHz)	f${}_{c}$ (MHz)	Dimensions (mm${}^{3}$)	Antenna Nr
800-DL	790–821	806	110 × 110 × 11	1
900-UL	879–915	896	100 × 100 × 11	2
900-DL	921–960	941	100 × 100 × 11	2
1800-UL	1710–1785	1748	90 × 100 × 6	3
1800-DL	1805–1880	1843	90 × 100 × 6	3
DECT	1880–1900	1890	90 × 100 × 6	3
2100-UL	1900–1980	1940	90 × 100 × 6	4
2100-DL	2110–2170	2140	90 × 100 × 6	4
WiFi-2G	2400–2485	2443	90 × 100 × 6	5
2600-DL	2620–2690	2655	90 × 100 × 6	5
WiFi-5G	5150–5875	5513	90 × 100 × 6	6

**Table 2:** Characteristics of the subjects participating in calibration measurements.

Subject	Sb-1	Sb-2	Sb-3	Sb-4	Sb-5	Sb-6
Gender	M	M	M	M	F	M
Age	28	43	61	39	39	30
Height (cm)	183	178	178	169	167	183
Weight (kg)	79	76	81	95	65	72
BMI (kg/m${}^{2}$)	23.6	23.9	25.5	33.2	23.3	21.5

**Table 3:** The median and $CI_{68}$ of PEMs’ responses on hips of Sb-1.

RF Signal	Sb-1: $p_{50}\left(R_{k}\right)$		Sb-1: $CI_{68}$ (dB)		${\mathrm{CI}}_{68}$ (dB) [30]
	EME Spy 200	ExpoM-RF	EME Spy 200	ExpoM-RF	ExpoM-RF
800-DL	0.17 ± 0.00	0.79 ± 0.020	9.28 ± 0.04	9.98 ± 0.10	12
900-UL	0.47 ± 0.008	0.3 ± 0.005	9.82 ± 0.15	9.35 ± 0.11	9.7
900-DL	0.37 ± 0.007	0.56 ± 0.017	9.57 ± 0.10	12.64 ± 0.12	9.4
1800-UL	0.23 ± 0.005	0.45 ± 0.010	11.04 ± 0.09	11.92 ± 0.03	12
1800-DL	0.19 ± 0.004	0.28 ± 0.003	10.95 ± 0.08	11.51 ± 0.11	13
DECT	0.12 ± 0.003	0.41 ± 0.000	11.67 ± 0.09	10.55 ± 0.09	13
2100-UL	0.26 ± 0.008	0.42 ± 0.006	10.94 ± 0.05	10.21 ± 0.04	13
2100-DL	0.27 ± 0.003	0.26 ± 0.005	12.91 ± 0.04	10.73 ± 0.03	12
WiFi-2G	0.34 ± 0.00	0.53 ± 0.009	12.52 ± 0.05	8.54 ± 0.03	14
2600-DL	0.73 ± 0.002	1.47 ± 0.008	8.99 ± 0.07	9.27 ± 0.03	18
WiFi-5G	0.41 ± 0.001	4.59 ± 0.039	13.68 ± 0.07	10.74 ± 0.08	15

**Table 4:** The optimized location and polarization of the nodes for BWDM and the median and $CI_{68}$ of the on-body AA.

RF Signal	${\mathrm{Pos}}^{\mathrm{pol}}$	$p_{50}\left(\mathrm{AA}\right)$ (cm${}^{2}$)		${\mathrm{CI}}_{68}$ (dB)		$\Delta_{{\mathrm{CI}}_{68}}$ dB (%)	
		Arithmetic	Geometric	Arithmetic	Geometric	EME Spy 200	ExpoM-RF
800-DL	$C^{H},Q^{V}$	16.95 ± 0.01	9.82 ± 0.04	5.42 ± 0.02	4.79 ± 0.04	−4.5 (93.7)	−5.2 (108.3)
900-UL	$I^{V},O^{H}$	20.88 ± 0.004	12.4 ± 0.2	4.43 ± 0.03	4.95 ± 0.03	−4.8 (98.3)	−4.4 (88.8)
900-DL	$G^{H},K^{V}$	26.43 ± 0.17	12.71 ± 0.13	3.9 ± 0.03	5.15 ± 0.04	−4.4 (85.8)	−7.5 (145.4)
1800-UL	$A^{V},S^{H}$	15.77 ± 0.07	4.35 ± 0.01	6.91 ± 0.04	3.79 ± 0.02	−7.2 (191.3)	−8.1 (214.5)
1800-DL	$D^{H},R^{V}$	10.73 ± 0.03	2.58 ± 0.01	6.61 ± 0.01	3.75 ± 0.03	−7.2 (192)	−7.7 (206.9)
DECT	$H^{H},L^{V}$	19.85 ± 0.002	4.44 ± 0.01	6.11 ± 0.01	4.53 ± 0.03	−7.1 (157.6)	−6 (132.9)
2100-UL	$F^{V},P^{H}$	13.33 ± 0.04	2.57 ± 0.01	8.47 ± 0.09	4.54 ± 0.02	−6.4 (141)	−5.7 (124.8)
2100-DL	$U^{H},V^{V}$	0.68 ± 0.003	0.34 ± 0.002	6.25 ± 0.03	3.3 ± 0.04	−9.6 (291.2)	−7.4 (225.1)
WiFi-2G	$J^{V},N^{H}$	12.93 ± 0.08	2.68 ± 0.007	7.05 ± 0.04	4.94 ± 0.02	−7.6 (153.4)	−3.6 (72.8)
2600-DL	$B^{H},T^{V}$	15.94 ± 0.07	2.92 ± 0.01	7.95 ± 0.02	5.67 ± 0.04	−3.3 (58.5)	−3.6 (63.5)
WiFi-5G	$E^{H},M^{V}$	1.01 ± 0.02	0.33 ± 0.003	11.58 ± 0.12	5.57 ± 0.03	−8.1 (145.6)	−5.1 (92.8)

**Table 5:** 68% confidence interval of the on-body antenna aperture for different subjects wearing the BWDM (combination of two antennas per frequency band).

RF Signal	${\mathrm{CI}}_{68}\;\pm \sigma$ (dB)					
	Sb-1	Sb-2	Sb-3	Sb-4	Sb-5	Sb-6
800-DL	4.79 ± 0.04	5.18 ± 0.08	4.43 ± 0.06	4.6 ± 0.01	4.23 ± 0.04	4.91 ± 0.06
900-UL	4.95 ± 0.03	5.53 ± 0.06	5.56 ± 0.03	4.75 ± 0.03	4.62 ± 0.02	5.09 ± 0.05
900-DL	5.15 ± 0.04	5.54 ± 0.04	5.87 ± 0.04	5.73 ± 0.08	5.33 ± 0.06	6.28 ± 0.06
1800-UL	3.79 ± 0.02	5 ± 0.04	5.76 ± 0.02	4.41 ± 0.02	5.35 ± 0.03	4.49 ± 0.04
1800-DL	3.75 ± 0.03	4.09 ± 0.02	5.59 ± 0.05	6.58 ± 0.03	7.35 ± 0.02	3.86 ± 0.02
DECT	4.53 ± 0.03	5.45 ± 0.01	6.38 ± 0.04	6.27 ± 0.06	5.71 ± 0.05	4.94 ± 0.01
2100-UL	4.54 ± 0.02	4.93 ± 0.09	5.08 ± 0.04	6.15 ± 0.06	5.77 ± 0.02	4.05 ± 0.03
2100-DL	3.3 ± 0.04	2.7 ± 0.01	3.59 ± 0.04	4.27 ± 0.03	3.3 ± 0.04	3.22 ± 0.03
WiFi-2G	4.94 ± 0.02	5.22 ± 0.04	5.50 ± 0.02	5.59 ± 0.04	4.84 ± 0.00	4.12 ± 0.02
2600-DL	5.67 ± 0.04	6 ± 0.02	5.49 ± 0.04	6.58 ± 0.03	6.34 ± 0.00	5.24 ± 0.03
WiFi-5G	5.57 ± 0.03	5.54 ± 0.05	5.89 ± 0.03	4.82 ± 0.08	8.79 ± 0.05	5.22 ± 0.06

**Table 6:** Studied frequency bands and their median on-body antenna apertures determined from calibration measurements on six subjects and uncertainties due to the presence of human body on the average of two nodes in each frequency band.

RF Signal	$p_{50}\left(\mathrm{AA}\right)$ (cm${}^{2}$)						$s_{\mathrm{low}}$ (%), $s_{\mathrm{up}}$ (%)					
	Sb-1	Sb-2	Sb-3	Sb-4	Sb-5	Sb-6	Sb-1	Sb-2	Sb-3	Sb-4	Sb-5	Sb-6
800-DL	9.8	9.2	10	7.7	9.3	14.1	42.4	47.6	35.7	43.1	39.6	33.2
							73.8	72.6	78.2	64.5	59.6	107.4
900-UL	12.4	13.1	10.3	9.4	10.3	6.8	40	47.7	43.5	47.5	45.6	41.7
							86.8	86.7	103.1	57	57.8	88
900-DL	12.7	5.3	8.45	11.8	10.4	5.9	41.1	50.3	47.9	43.5	46.2	46.5
							92.4	78.3	101.7	111.6	83.6	126.6
1800-UL	4.35	4.7	3.8	4	3.5	4.6	33.9	36.6	47.4	33	46	40.7
							58.4	100.4	97.2	85.2	84.8	67.1
1800-DL	2.58	2.2	2.1	1.8	1.9	3.8	28.1	40.6	52.4	57.3	63.9	29.6
							70.3	52.2	72.3	94.2	96	71.5
DECT	4.44	2.93	3.9	4	3.8	3.9	33.6	46.9	54.9	48.2	40.2	46.6
							88.6	86.3	96.1	119.4	122.3	66.3
2100-UL	2.57	2.5	1.8	1.6	2.3	2.3	29.1	36.8	44.7	48.4	45.8	35.1
							102	97.3	78.6	113	104.9	65
2100-DL	0.34	0.34	0.33	0.29	0.31	0.2	27.8	26.8	34.2	39.4	27.3	35.4
							54.7	36.3	50.4	62.4	55.3	35.6
WiFi-2G	2.68	3.13	2.38	2	3	2.2	39.6	44	55	48.3	45.9	36.4
							88.3	86.2	60	87.5	65.2	64.2
2600-DL	2.92	2.7	3.1	3.4	4.2	4.5	41.8	43.9	46.9	44.6	50.2	43.7
							115	122.8	88	152.2	114.6	88.2
WiFi-5G	0.33	0.39	0.34	0.42	0.51	0.36	39.2	47.5	45.7	31.5	52.2	44.3
							119.4	88.1	110.9	107.8	262.3	85
$s_{low,total}$							36.1	42.6	46.7	45.7	45.8	39.2
$s_{up,total}$							84.9	85.1	84.2	98.8	87.5	81.6

**Table 7:** Summary statistics of four downlink bands measured during the walk for the BWDM and two conventional PEMs.

RF Signal	Device	$S_{inc}$ (μW/m${}^{2}$)						
		μ	σ	$p_{25}$	$p_{50}$	$p_{75}$	Ratio $p_{50}$	Censored Data (%)
800-DL	BDWM	26.7	101	1.14	3.21	14.3	-	-
	EME Spy 200	40.73	298	0.26	1.28	10.8	2.5	2.52
	ExpoM-RF	52.4	240	0.55	1.91	20.75	1.68	-
900-DL	BDWM	90.8	226	14	28	78.6	-	-
	EME Spy 200	60.1	233	5.3	12.26	39.3	2.28	-
	ExpoM-RF	151.5	404	13.62	34.77	105.4	0.35	-
1800-DL	BWDM	37.7	82	5.46	12.2	38.6	-	-
	EME Spy 200	4.41	11.4	0.16	0.59	3.24	20.67	13.47
	ExpoM-RF	14.53	33	0.45	1.62	13.5	7.4	1.03
2100-DL	BWDM	69.8	107	20.6	29	65.1	-	4.2
	EME Spy 200	26.2	104	1	3.06	12.26	9.47	0.63
	ExpoM-RF	23.9	71.3	0.98	3.15	13.97	9.2	-

## Abbreviations

The following abbreviations are used in this manuscript:

BWDM	multi-band Body-Worn Distributed exposure Meter
PEM	Personal Exposure Meters
RF	Radio-Frequency
PDE	Personal Distributed Exposure meter
BMI	Body Mass Index
UL	Uplink
DL	Downlink
GSM	Global System for Mobile Communications
UMTS	Universal Mobile Telecommunications System
DECT	Digital Enhanced Cordless Telecommunications
WiFi	Wireless Fidelity
LTE	Long-Term Evolution
SIW	Substrate Integrated Waveguide technology
AA	Antenna Aperture
RX	Receiver Node
TX	Transmitting Antenna

## References

[1] Ahlbom A., Bergqvist U., Bernhardt J.H., Cesarini J.P., Grandolfo M., Hietanen M., Mckinlay A.F., Repacholi M.H., Sliney D.H., Stolwijk J.A. (1998). Guidelines for limiting exposure to time-varying electric, magnetic, and electromagnetic fields (up to 300 GHz). Health Phys..

[2] Röösli M., Frei P., Mohler E., Braun-Fahrländer C., Bürgi A., Fröhlich J., Neubauer G., Theis G., Egger M. (2008). Statistical analysis of personal radiofrequency electromagnetic field measurements with nondetects. Bioelectromagnetics.

[3] De Miguel-Bilbao S., García J., Ramos V., Blas J. (2015). Assessment of human body influence on exposure measurements of electric field in indoor enclosures. Bioelectromagnetics.

[4] Gryz K., Zradziński P., Karpowicz J. (2015). The role of the location of personal exposimeters on the human body in their use for assessing exposure to the electromagnetic field in the radiofrequency range 98–2450 MHz and compliance analysis: Evaluation by virtual measurements. BioMed Res. Int..

[5] Bolte J.F., van der Zande G., Kamer J. (2011). Calibration and uncertainties in personal exposure measurements of radiofrequency electromagnetic fields. Bioelectromagnetics.

[6] Thielens A., Agneessens S., Verloock L., Tanghe E., Rogier H., Martens L., Joseph W. (2015). On-body calibration and processing for a combination of two radio-frequency personal exposimeters. Radiat. Prot. Dosim..

[7] Thielens A., Agneessens S., De Clercq H., Lecoutere J., Verloock L., Tanghe E., Aerts S., Puers R., Rogier H., Martens L. (2015). On-body calibration and measurements using a personal, distributed exposimeter for wireless fidelity. Health Phys..

[8] Neubauer G., Cecil S., Giczi W., Petric B., Preiner P., Fröhlich J., Röösli M. (2010). The association between exposure determined by radiofrequency personal exposimeters and human exposure: A simulation study. Bioelectromagnetics.

[9] Thielens A., De Clercq H., Agneessens S., Lecoutere J., Verloock L., Declercq F., Vermeeren G., Tanghe E., Rogier H., Puers R. (2013). Personal distributed exposimeter for radio frequency exposure assessment in real environments. Bioelectromagnetics.

[10] Bolte J.F., Eikelboom T. (2012). Personal radiofrequency electromagnetic field measurements in the Netherlands: Exposure level and variability for everyday activities, times of day and types of area. Environ. Int..

[11] Valič B., Kos B., Gajšek P. (2015). Typical exposure of children to EMF: Exposimetry and dosimetry. Radiat. Prot. Dosim..

[12] Gajšek P., Ravazzani P., Wiart J., Grellier J., Samaras T., Thuróczy G. (2014). Electromagnetic field exposure assessment in Europe radiofrequency fields (10 MHz–6 GHz). J. Expo. Sci. Environ. Epidemiol..

[13] Mann S. (2010). Assessing personal exposures to environmental radiofrequency electromagnetic fields. C. R. Phys..

[14] Joseph W., Frei P., Roösli M., Thuróczy G., Gajsek P., Trcek T., Bolte J., Vermeeren G., Mohler E., Juhász P. (2010). Comparison of personal radio frequency electromagnetic field exposure in different urban areas across Europe. Environ. Res..

[15] Röösli M., Frei P., Bolte J., Neubauer G., Cardis E., Feychting M., Gajsek P., Heinrich S., Joseph W., Mann S. (2010). Conduct of a personal radiofrequency electromagnetic field measurement study: Proposed study protocol. Environ. Health.

[16] Iskra S., McKenzie R., Cosic I. (2011). Monte Carlo simulations of the electric field close to the body in realistic environments for application in personal radiofrequency dosimetry. Radiat. Prot. Dosim..

[17] Bolte J.F. (2016). Lessons learnt on biases and uncertainties in personal exposure measurement surveys of radiofrequency electromagnetic fields with exposimeters. Environ. Int..

[18] Blas J., Lago F.A., Fernández P., Lorenzo R.M., Abril E.J. (2007). Potential exposure assessment errors associated with body-worn RF dosimeters. Bioelectromagnetics.

[19] Bahillo A., Blas J., Fernández P., Lorenzo R.M., Mazuelas S., Abril E.J. (2008). E-field assessment errors associated with RF dosemeters for different angles of arrival. Radiat. Prot. Dosim..

[20] De Miguel-Bilbao S., Ramos V., Blas J. (2017). Assessment of polarization dependence of body shadow effect on dosimetry measurements in 2.4 GHz band. Bioelectromagnetics.

[21] Aminzadeh R., Thielens A., Bamba A., Kone L., Gaillot D.P., Lienard M., Martens L., Joseph W. (2016). On-body calibration and measurements using personal radiofrequency exposimeters in indoor diffuse and specular environments. Bioelectromagnetics.

[22] López A.N., Gonzalez-Rubio J., Montoya J.M.V., Garde E.A. (2015). Using multiple exposimeters to evaluate the influence of the body when measuring personal exposition to radio frequency electromagnetic fields. COMPEL.

[23] Vanveerdeghem P., Van Torre P., Thielens A., Knockaert J., Joseph W., Rogier H. (2015). Compact personal distributed wearable exposimeter. IEEE Sens. J..

[24] Aminzadeh R., Thielens A., Li H., Leduc C., Zhadobov M., Torfs G., Bauwelinck J., Martens L., Joseph W. (2017). Personal Exposimeter for Radiation Assessment in Real Environments in the 60-GHz Band. Radiat. Prot. Dosim..

[25] Agneessens S. (2017). Coupled eighth-mode Substrate Integrated Waveguide Antenna: Small and Wideband with High-Body Antenna Isolation. IEEE Access.

[26] Neubauer G., Cecil S., Giczi W., Petric B., Preiner P., Frolich J., Röösli M. (2008). Evaluation of the Correlation between RF Dosimeter Reading and Real Human Exposure.

[27] Agneessens S., Rogier H. (2014). Compact Half Diamond Dual-Band Textile HMSIW On-Body Antenna. IEEE Trans. Antennas Propag..

[28] Thielens A., Vanveerdeghem P., Van Torre P., Gängler S., Röösli M., Rogier H., Martens L., Joseph W. (2016). A Personal, Distributed Exposimeter: Procedure for Design, Calibration, Validation, and Application. Sensors.

[29] Belgian Institute for Postal Services and Telecommunications. http://www.bipt.be.

[30] Bhatt C.R., Thielens A., Billah B., Redmayne M., Abramson M.J., Sim M.R., Vermeulen R., Martens L., Joseph W., Benke G. (2016). Assessment of personal exposure from radiofrequency-electromagnetic fields in Australia and Belgium using on-body calibrated exposimeters. Environ. Res..

[31] Balanis C.A. (2005). Antenna Theory: Analysis and Design.

[32] Bhatt C.R., Thielens A., Redmayne M., Abramson M.J., Billah B., Sim M.R., Vermeulen R., Martens L., Joseph W., Benke G. (2016). Measuring personal exposure from 900 MHz mobile phone base stations in Australia and Belgium using a novel personal distributed exposimeter. Environ. Int..

[33] Hardell L., Carlberg M., Koppel T., Hedendahl L. (2017). High radiofrequency radiation at Stockholm Old Town: An exposimeter study including the Royal Castle, Supreme Court, three major squares and the Swedish Parliament. Mol. Clin. Oncol..

